# A 12-month weight loss intervention in adults with obstructive sleep apnoea: is timing important? A step wedge randomised trial

**DOI:** 10.1038/s41430-022-01184-5

**Published:** 2022-08-04

**Authors:** Helen Truby, Bradley A. Edwards, Kaitlin Day, Denise M. O’Driscoll, Alan Young, Ladan Ghazi, Claire Bristow, Kerryn Roem, Maxine P. Bonham, Chiara Murgia, Terry P. Haines, Garun S. Hamilton

**Affiliations:** 1 School of Human Movement and Nutrition Sciences, University of Queensland, Melbourne, VIC Australia; 2 Department of Physiology, Biomedicine Discovery Institute, Monash University, Melbourne, VIC Australia; 3 School of Psychological Sciences and Turner Institute for Brain and Mental Health, Monash University, Melbourne, VIC Australia; 4 School of Clinical Sciences, Department of Nutrition, Dietetics and Food, Monash University, Melbourne, VIC Australia; 5 Eastern Health, Department of Respiratory and Sleep Medicine, Melbourne, VIC Australia; 6 Eastern Health Clinical School, Monash University, Melbourne, VIC Australia; 7 School of Public Health and Preventive Medicine, Monash University, Melbourne, VIC Australia; 8 School of Agriculture and Food, Melbourne University, Melbourne, VIC Australia; 9 School of Primary and Allied Health Care, Monash University, Melbourne, VIC Australia; 10 School of Clinical Sciences, Monash University, Melbourne, VIC Australia; 11 Monash Health, Department of Lung, Sleep, Allergy and Immunology, Melbourne, VIC Australia

**Keywords:** Obesity, Weight management

## Abstract

**Background/Objectives:**

Continuous positive airway pressure (CPAP) concomitant with weight loss is a recommended treatment approach for adults with moderate-severe obstructive sleep apnoea (OSA) and obesity. This requires multiple synchronous behaviour changes. The aim of this study was to examine the effectiveness of a 6-month lifestyle intervention and to determine whether the timing of starting a weight loss attempt affects weight change and trajectory after 12 months in adults newly diagnosed with moderate-severe OSA and treated at home with overnight CPAP.

**Methods:**

Using a stepped-wedge design, participants were randomised to commence a six-month lifestyle intervention between one and six-months post-enrolment, with a 12-month overall follow-up. Adults (*n* = 60, 75% males, mean age 49.4 SD 10.74 years) newly diagnosed with moderate-severe OSA and above a healthy weight (mean BMI 34.1 SD 4.8) were recruited.

**Results:**

After 12 months, exposure to the intervention (CPAP and lifestyle) resulted in a 3.7 (95% CI: 2.6 to 4.8, *p* < 0.001) kg loss of weight compared to the control condition (CPAP alone). Timing of the weight loss attempt made no difference to outcomes at 12 months. When exposed to CPAP only (control period) there was no change in body weight (Coef, [95% CI] 0.03, [−0.3 to 0.36], *p* = 0.86).

**Conclusions:**

The lifestyle intervention resulted in a modest reduction in body weight, while timing of commencement did not impact the degree of weight loss at 12 months. These findings support the recommendation of adjunctive weight-loss interventions within six-months of starting CPAP.

## Introduction

Obesity and Obstructive Sleep Apnoea (OSA) share a complex and bi-directional relationship making simultaneous treatment of both at an individual level, challenging [1, 2]. Excess weight, in particular in the abdominal and upper airway regions, contributes both to OSA aetiology and progression of symptoms [3]. The fragmented sleep associated with OSA may contribute to reinforcing the obese state due to adverse alterations in eating behaviours including increased appetite as well as reductions in both satiety and physical activity [1].

The first line treatment in adults newly diagnosed with moderate-severe OSA is continuous positive airway pressure treatment (CPAP) [4]. However, adherence after CPAP initiation can decline rapidly, with non-adherence rates as low as 38% six-months after treatment initiation, and individual mean usage as low as 50% in the long term [5, 6]. Evidence from systematic reviews and meta-analyses have concluded that lifestyle interventions can improve OSA severity with a mean reduction in apnoea-hypopnea index (AHI) and BMI of 9 events/hr and 2.46 kg/m^2^, respectively [7, 8]. As such, current guidelines recommend weight loss in conjunction with CPAP use for the treatment of OSA in those above a healthy weight [9]. In 2019, a meta-analysis revealed only three randomised controlled trials which had assessed the efficacy of CPAP in conjunction with a lifestyle intervention in adults with moderate-severe OSA and obesity [7]. Whilst reporting reductions in both AHI (−8.7 events/hr) and BMI (−3.5 kg/m^2^), two of the studies were of short duration (≤9 weeks) and none included a post-intervention follow-up period [10–12].

Lifestyle modification requires individuals to engage and undertake different, but complex behavioural processes to lose weight. Behaviour change theory states that whilst people can be motivated to change multiple behaviours at once, actioning multiple changes simultaneously increases the risk of failure [13, 14]. Individuals newly diagnosed with OSA, are faced with a multitude of recommended changes to their lifestyle that they are asked to synchronously adopt. Initiation of a lifestyle intervention alongside CPAP treatment, according to behaviour change theory, is likely to result in delayed adoption of one of these behaviours [13]. Within this context, the optimal timing to initiate lifestyle change in those newly diagnosed with OSA and commencing on CPAP is unknown. As it would be unethical to withhold effective treatments in those with known risk factors for cardiovascular disease, we deployed a stepped-wedge randomised controlled trial design which allows each participant to receive the interventions of interest but in a staggered approach to exposure [15]. As such, we aimed to investigate (1) the effectiveness of a six-month lifestyle intervention for reducing body weight in overweight subjects with moderate-severe OSA commencing CPAP and (2) whether the timing of commencement of a lifestyle intervention affects the change in weight and trajectory of weight experienced 12 months following CPAP commencement. Secondary aims were to (1) Examine whether people who commence CPAP gain body weight and (2) To explore whether offering participants a smartphone App to maintain contact with their health professional could improve outcomes by supporting small changes in behaviour over 12 months.

## Methods

This prospectively registered trial (Australia and New Zealand clinical trial registry Number: ACTRN12616000203459) was reviewed by the relevant Ethical institutional review boards and written informed consent was obtained from participants. A detailed methodology paper has been published [16]. Briefly, 60 adults aged between 19–68 years were recruited with a BMI above the healthy range (>25–43 kg/m^2^ for Caucasians and in those of Asian or Indian descent BMI > 23 kg/m^2^); who were newly diagnosed with moderate-severe OSA (AHI > 20 events/hour [17]) and who were recommended CPAP therapy. Overnight polysomnography was completed at screening and after 12 months to determine AHI. Specific details of the polysomnography are included in the Supplementary file.

### Design

**Fig. 1:**
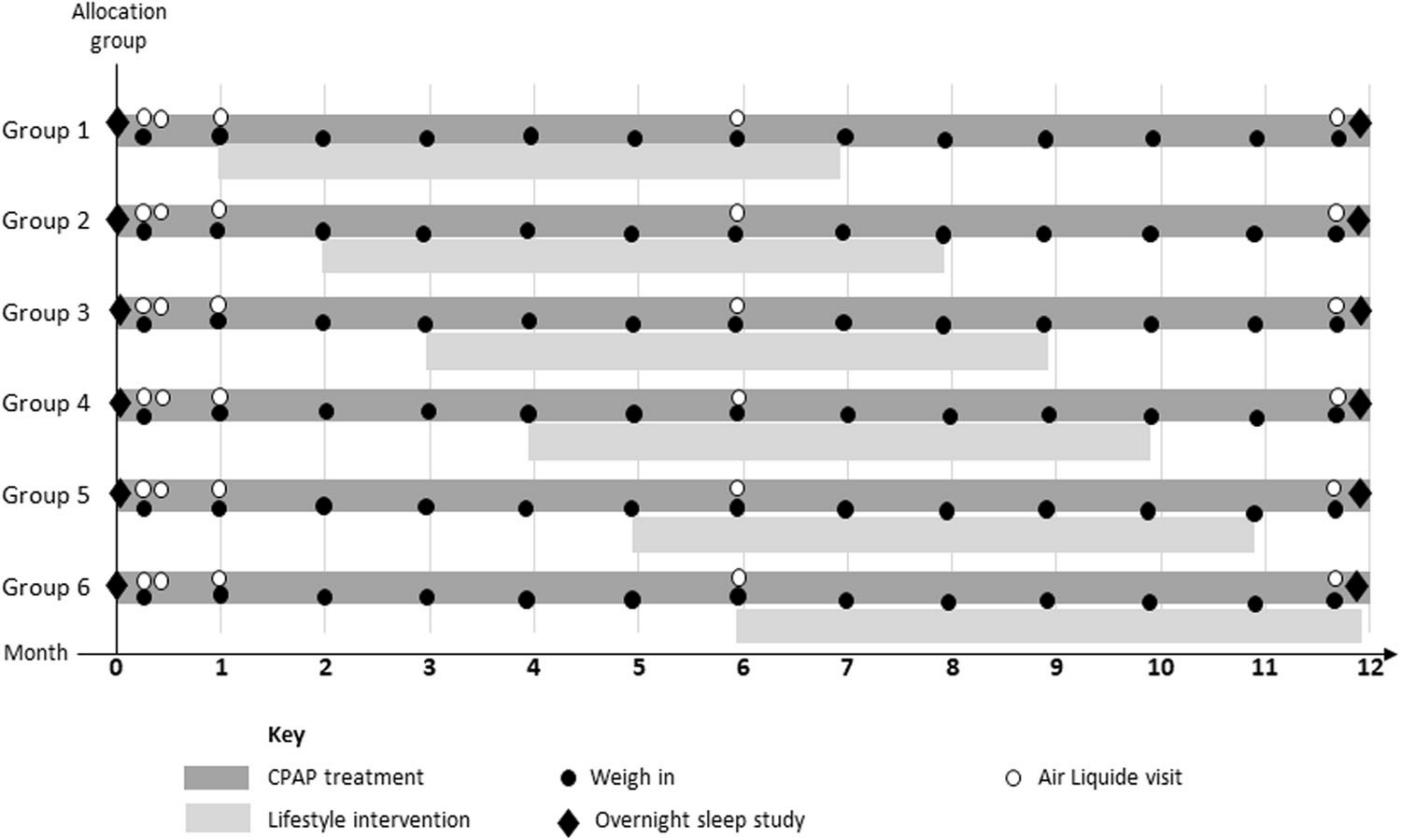
Study design of The Sleeping Well Trial. A cluster-randomised stepped-wedge design. The 6 groups allocated to a delayed lifestyle intervention start of between 1–6 months. Symbols represent data collection points. CPAP treatment, lifestyle intervention, weight measurement, overnight polysomnography, visit to Air Liquide centre for CPAP support.

A stepped-wedge, randomised control design (Fig. 1) was implemented [15]. This study design was chosen because stepped-wedge randomised trials involve within-cluster comparisons between time periods when participants are and are not exposed to an intervention. Thus, when examining when the lifestyle intervention had an effect on change in weight, comparisons can be made between different time periods within groups. Within group contrasts offer substantial statistical power advantages compared to between group contrasts thus our trial could be constructed with a smaller overall sample size than what may be present in conventional trials. Randomisation occurred after enrolment (simple randomisation, implemented using Microsoft Excel by the study statistician (TH)). These were stored in opaque envelopes until after recruitment and the baseline assessment was completed, when they were revealed to the researcher and the patient at the same time who then knew how long they would wait for their weight loss intervention to commence. Participants were asked not to reveal their group allocation to their treating CPAP technicians or sleep physician who were blinded to group allocation. Visits for data collection at the university were staggered by appointment time, thus minimising risk of participants mingling. All participants undertook one month of CPAP alone and then subjects commenced their lifestyle intervention at the end of month one through to month six.

### Polysomnography

Full Polysomnography was performed in an in-patient setting (level 1) or in the patient’s home (level 2). The recording included electroencephalogram (EEG), bilateral electrooculogram (EOG), mentalis/submentalis electromyogram (EMG), anterior tibialis (left and right) EMG and electrocardiogram (ECG). Respiration was assessed via nasal pressure cannula + /− oronasal thermistor, thoracic and abdominal respiratory inductance plethysmography (RIP) bands, and fingertip pulse oximetry.

Sleep stages, arousal, and respiratory events were scored according to American Academy of Sleep Medicine (AASM) 2012 recommended criteria using Profusion PSG3 software (Compumedics, Abbotsford, Victoria, Australia) [17]. Specifically, AHI was defined as the total number of apnoea’s and hypopneas per hour of sleep. Apnoea was defined as a ≥ 90% decrease in oronasal airflow for at least 10 s. Hypopneas were scored when nasal pressure signal dropped by ≥30% from baseline for ≥10 s and the event was associated with either a 3% or greater fall in oxygen and/or an arousal from sleep.

### CPAP

CPAP treatment was provided centrally for all participants by Air Liquide Healthcare (Melbourne, Australia). For the first week, participants were commenced on an auto titrating CPAP and afterwards were switched to a fixed pressure based on the 95th centile pressure. Routine care and decisions regarding treatment changes were provided by the participants’ treating physician, who were blinded to group allocation.

### Intervention

Participants were prescribed an intermittent energy restricted dietary pattern, which consisted of 5 days of restricted daily energy intake of 6300–7500 kilojoules (kJ) per day and were provided with meal plans to support their food selections and on 2 days per week, a very low energy intake (2200–2760 kJ per day) was prescribed by providing participants with a powered milk-shake that they made up at home along with multivitamins with fish oil, fibre and iron (women only). Participants were encouraged to eat low starch vegetables/salads and low joule fluids ad libitum. This procedure ensured overall good diet quality including micro-nutrient adequacy on the ‘fasting’ days [16]. One-to-one monthly appointments with the study dietician (KR) started when the participants were eligible to commence the active weight loss phase. For the duration of the six-month intervention, the dietician provided structured advice grounded in Michie’s behaviour change theory [18], with months one to three focusing on active weight loss, whilst months four to six focused on strategies to maintain lost weight. At the end of the 6-months of the intervention, participants were discharged from the study clinic having been prepared with strategies to maintain their weight on their own.

Participants were offered the use of a smartphone app (MyPace™) for communication with the dietician in between their monthly face-to-face appointments. On this App, participants could set and monitor their progress towards their own goals, track their weight loss and receive motivational texts. Participants were advised to increase their physical activity levels (i.e. 3 × 30 min/week moderate activity such as walking or swimming, a total of 90 min per week) by the dietician at their first appointment and were provided with a Fitbit^©^ to help participants’ monitor their own physical activity levels. Participants provided separate consent for the research team to access their individual web based FitBit^©^ account to monitor usage and activity. App usage and physical activity are provided in the Supplementary file.

### Primary outcome measure

Weight to the nearest 0.1 kg was measured monthly (SECA Clara 803) without shoes.

### Statistical analysis

A detailed description of the statistical analysis protocol has been previously described [16] with further detail provided in the supplementary file. Multilevel, mixed effects generalized linear models were used to investigate the primary aims. These linear mixed models include all participants with data regardless of whether all follow-up data points were collected or not. Our primary analysis compared weight measurements taken during the intervention period of the stepped wedge, to those taken during the earlier control period. The planned approach was to undertake this contrast using one “change in weight (T_n_ minus T_n-1_)” measurement for each participant for each month of the stepped wedge trial. A further analysis used data from both the stepped wedge portion of the design and the 12-month follow-up measurement. Participant number was treated as a random effect to account for dependency of observations within each participant, a complete case analysis was performed, and an intention-to-treat approach employed. A categorical fixed effect for month since trial commencement was included to account for temporal trends [15, 19]. Primary aim 2) was examined using analysis two, but with the addition of an intervention-by-month of commencement interaction effect. Primary aim 2) was also investigated using an ANCOVA-style linear regression approach (analysis three) using only the final assessment of weight treated as the dependent variable, the first weight measurement as a covariate, and month of commencement of the intervention as a categorical independent variable. Treating month of commencement as a linear variable + /− transformations was explored if analyses two & three revealed a linear, quadratic or other trend in the effect of month of commencement on the dependent variable (analysis four).

Secondary aim 1) was assessed using only pre-intervention period data from the stepped wedge portion of the trial using a multi-level, mixed effects, generalized linear model (analysis five). Trial participants were treated as a random effect.

### Power calculation and sample size

Recruiting seven participants for each of the six starting time points of the lifestyle intervention provided 90% power to detect a standardised effect size of 0.40 for the rate of change in body weight outcome for the primary analysis. This assumes a conservative intraclass correlation coefficient of 0.10, two-tailed α = 0.05, and treats each individual participant as its own cluster (as this is the unit of randomisation). We collected 10 participants per group to maintain trial power allowing for a potential drop-out/missing data rate of over 30%.

Treating physicians were blinded to group allocation but participants were not due to the necessity for them to undertake the weight loss intervention.

### Variation to study analysis protocol

We had intended to use an individual’s monthly average CPAP treatment adherence since the previous assessment as a covariate in analyses one to four, and for contamination-adjusted intention-to-treat analyses (planned analyses six to eight). However, data extracted from CPAP machines used in this trial were captured as summative across the trial period and not on a month-by-month basis, preventing us from being able to use this variable for these analyses. Planned analyses six to eight described in the protocol were not undertaken. One participant was recruited into the study but after further analysis of the overnight sleep study, was found to have only mild OSA and was therefore excluded from all analyses.

We undertook a post-hoc sensitivity analysis for comparisons of weight between intervention and control periods using raw weight as the dependent variable instead of change in weight from the previous assessment to assist with interpretation of the effect size estimates generated. We examined whether those who continued use of CPAP had different mean weight scores at 12-month follow-up compared to those who did not commence or discontinued CPAP, adjusted for weight at the baseline assessment using linear regression.

## Results

### Sample demographics

**Fig. 2:**
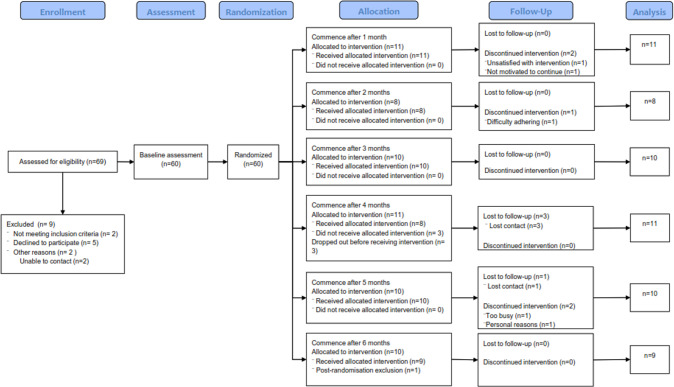
Consort flow diagram. Consort flow diagram of participants progress through The Sleeping Well Trial.

Sixty participants were recruited from March 2016 to May 2018 (Fig. 2). Fifty-nine were included in the analyses of which, 44 (75%) were men. Baseline characteristics including ethnicity and medication usage of the participants are provided in Table 1. Baseline and 12-month anthropometric data and AHI are presented in Table 2.

**Table 1:** Baseline characteristics of participants. Data presented as mean (SD) unless otherwise stated.

Age, years	49.4 (10.74)
Sex (male, %)	44 (75)
BMI (kg/m^2^)	34.0 (4.6)
Ethnicity (*n*, %)	
Caucasian	35 (59.32)
Asian, Chinese	5 (8.47)
Asian, Indian	5 (8.47)
Other	10 (16.95)
Co-mobidities (*n*, %)	
High cholesterol	6 (10.17)
Type 2 Diabetes	2 (3.39)
Hypertension	14 (23.73)
Asthma	7 (11.86)
Hypothyriodism	2 (3.39)
Other	18 (30.51)
Medications (*n*, %)	
Anti-depressives	12 (20)
Anti-hypertensives	14 (23.7)

**Table 2:** BMI and Apnea-hypopnea index (AHI) at baseline and at 12months.

	Baseline – total sample (*n* = 59)	Baseline	12 months follow-up	Mean difference* (95% CI), *p*-value
		In those who completed 12-month follow-up (*n* = 47 for weight and BMI, *n* = 39 for AHI)		
Weight (kg)	101.8 (21.9)	102.3 (21.8)	98.6 (21.7)	3.7 (1.6–5.8), *p* < 0.001
BMI (kg/m^2^)	34.1 (4.8)	34.2 (5.0)	33.0 (5.2)	1.2 (0.5–1.9), *p* < 0.001
AHI (events/hr)	46.9 (21.1)	43.6 (19.9)	36.1 (22.5)	7.5 (1.2–13.8), *p* = 0.02
ODI 3% (events/hr)	39.7 (22.7)	40.2 (23.1)	28.2 (21.3)	12.0 (5.4–18.7), *p* < 0.001
OSA classification – *n* (%)				
Normal	0 (0%)	0 (0%)	1 (2.6%)	
Mild OSA (AHI ≥5 to <15)	0 (0%)	0 (0%)	7 (18.0%)	
Mod. OSA (AHI ≥15 to <30)	17 (28.8%)	13 (33%)	12 (30.8%)	
Severe OSA (AHI ≥ 30)	42 (71.2%)	26 (66%)	19 (48.7%)	

Data presented as mean (SD) unless otherwise specified.

*AHI* apnea hypopnea index, *ODI* oxygen desaturation index as measured by overnight polysomnography, *BMI* body mass index, *OSA* obstructive sleep apnea.

*Mean difference equals baseline minus 12-month follow-up value.

### Primary and secondary aims

Participants recorded lower weight following exposure to the lifestyle intervention compared to beforehand during the stepped-wedge trial (Table 2). This was consistent with findings generated when the 12-month follow-up was included in the analysis. This was also consistent across our a-priori (using change in weight) and post-hoc sensitivity analyses (using raw weight). The timing of commencement of the lifestyle intervention had no effect on either rate of weight change or final weight at 12 months (no significant interaction effect between start month and intervention effect, *p* > 0.05, see Supplementary Tables 1, 3). Quadratic and other transformations of the ‘months waited until commencement of the lifestyle intervention’ variable did not impact this result (*p* > 0.05, see Supplementary Table 2). In summary, how long a participant waited to commence the lifestyle intervention did not affect their weight at 12-months follow up (all *p* values > 0.05, see Supplementary Table 3).

**Fig. 3:**
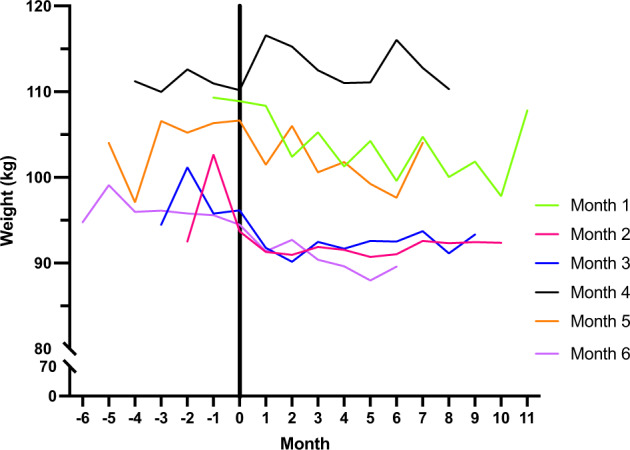
Line graph of monthly weight data for The Sleeping Well Trial. Each line represents a group, who were randomised to begin the 6-month lifestyle intervention between 1- and 6-months post-enrolment. The black line at 0 represents the start of the lifestyle intervention for all participants. Monthly weight is calculated as the average for each group. All participants were followed for 12-months and received the 6-month lifestyle intervention during this period.

A graph displaying summative weight values for each time period across the groups is displayed in Fig. 3.

Raw weight measures in participants when they were exposed to CPAP alone were not significantly different between the baseline assessment (before commencement of CPAP) and after commencement of CPAP (but before commencement of the lifestyle intervention -Coef [95% CI]: 0.13 [−0.55 to 0.81], *p* = 0.71).

### Interpretation of analyses addressing primary and secondary aims

The interpretation of coefficients obtained in an a-priori analyses examining the effect of the lifestyle intervention on weight indicated a 1.8 kg greater loss in weight per month after exposure to the intervention. This means we might expect to see at least a 11 kg weight loss overall in a trial where each participant had a minimum of six months following exposure to the lifestyle intervention. However, the raw data indicated that of the *n* = 47 participants with weight measurements at baseline and 12-month follow-up, the mean (SD) weight at baseline was 102.3 kgs, and at 12-month follow-up was 98.6 kgs. The difference (95% CI) between these was 3.7 (1.6 to 5.8) kgs (*p* < 0.001). Additionally, the mean (SD) change in weight in the first month following commencement of the lifestyle intervention was a loss of 2.6 (2.5) kgs (*n* = 45), in the second month was a loss of 1.4 (1.7) kgs (*n* = 41), in the third month was a loss of 1.3 (1.7) kgs (*n* = 42), and in the fourth month was a loss of 0.8 (1.4) kgs (*n* = 41). Following this the mean monthly weight changes ranged between a gain of 0.7 kgs and a loss of 0.4 kgs. In contrast to the a-priori analyses, the interpretation of coefficient obtained in the post-hoc sensitivity analyses using raw weight as the outcome instead of change in weight (T_n_ – T_n-1_) indicated that means of raw weight measures collected each month following commencement of the lifestyle intervention were 2.7 (95% CI: 1.7–3.6) kgs lower (stepped-wedge component only) and 3.7 (95% CI: 2.6–4.8) kgs lower (whole trial) compared to measures taken before commencement of the intervention. These findings create an interpretation that is more consistent with the raw data and provide the most useful indicator of effect size.

### AHI change

Baseline screening AHI values (*n* = 59, mean = 46.9, SD = 21.1) were higher than 12-month follow-up AHI (*n* = 39, mean = 36.1, SD = 22.6). A comparison across these time points identified that the mean 12-month follow up AHI was 10.75 units lower (95% CI −17.6 to −3.87, *p* = 0.003) than at baseline. There was a reduction in the proportion of participants classified as having severe OSA in post-trial polysomnography (*n* = 20 of 40 tested, 50%), compared to baseline (*n* = 42 of 59 tested, 71%). (See Table 3). There was no impact of month of commencement of the lifestyle intervention on AHI severity at 12 months, after adjusting for baseline (regression co-efficient (95% CI) −0.70 (−4.1 to 2.75), *p* = 0.68).

**Table 3:** Outcome of a-priori and post-hoc, sensitivity analyses examining effect of exposure to lifestyle intervention on weight outcomes (primary aims 1 and 2).

Analysis	A-priori	A-priori	Post-hoc, sensitivity	Post-hoc, sensitivity
Outcome variable	Monthly change in weight	Monthly change in weight	Raw weight	Raw weight
Time-frame	Stepped-wedge only	Stepped-wedge and 12-month follow-up	Stepped-wedge only	Stepped-wedge and 12-month follow-up
Coefficient (exposure to lifestyle intervention)	1.8 (favors intervention) kg loss per month more during intervention period than control period	1.9 (favors intervention) kg loss per month more during intervention period than control period	−2.7 (favors intervention) difference in mean weight measurements during intervention period than control period	−3.7 (favors intervention) difference in mean weight measurements during intervention period than control period
95% CI	1.2–2.4	1.3–2.5	−1.7 to −3.6	−2.6 to −4.8
*p*-value	<0.001	<0.001	<0.001	<0.001
ICC	0.07	0.06	0.98	0.97
Observations	297	492	386	600
Participants	56*	56*	59	59

*Note that three participants only had the baseline assessment and could therefore not generate a monthly change in weight score.

### Adherence

Intervention dose overall was high, with the median number of sessions attended 14 out of 16 (87.5%) (dietitian 6 out of 6 (100%), Air Liquide™ 1 out of 3 (33%) and research centre 7 out of 7 (100%)). Supplementary Fig. E1 presents an overview of participant monthly attendance over the trial period. Intervention fidelity, the extent to which the intervention was delivered as planned was moderate-to- high, with the dietary intervention protocol being delivered successfully in accordance with the protocol developed pre-trial. Exceptions existed when participants’ developed conditions in which variances in procedures needed to occur, i.e., developing type 2 diabetes, irritable bowel syndrome (IBS) or sustaining an injury. In such instances, other arrangements were made (e.g. follow a low FODMAP diet whilst maintaining two fasting days, practice rehabilitation exercises) whilst trying to adhere to the original protocol as closely as possible.

### Physical activity and Fitbit^©^

The median number of months in which participants used the Fitbit^©^ device (defined as at least one recording present on a participant’s online accounts) was 10 months (IQR 5, 12). Months four, nine and ten were the most frequent months in which usage of the device ceased. Participants over 50 years of age engaged with the device more than those under 50 years (*p* < 0.001).

### Engagement with smartphone app component of intervention

This study sought to explore the potential of a smartphone app (MyPace^TM^) to improve adherence with interventions, both lifestyle and with self-administered CPAP and extend the reach and impact of face-to-face advice which is the traditional model of care in hospitals. Forty-one out of 47 completers had the MyPace™ application installed on their smartphone devices. The remaining participants did not have a smartphone on which the App could be installed. Twenty-three (49%) participants did not interact with the dietician at all using MyPace™, hence the impact of this aspect of the trial was low. There was no difference in MyPace™ usage amongst age groups. User ratings were ranked on a Likert scale (1 = very unhelpful and 5 = very helpful), with the median rating being 2.0 (IQR: 1.0–3.0). See the supplementary file for the process evaluation.

### Participant engagement/adherence with use of CPAP

For the CPAP intervention, 40 participants used CPAP across the trial. One of these participants had a faulty machine preventing download of adherence data but was known to have continued with CPAP use across the trial. Twelve-month weight data were available for 38 participants who used CPAP. There were nine participants who did not commence or ceased using CPAP but did provide 12-month weight data. There was no difference in 12-month weight between those who used CPAP across the trial (*x* = 1) and those who did not commence or ceased using CPAP (*x* = 0) after adjusting for baseline weight [Coef (95% CI), *p*-value: 0.2 (−5.1 to 5.6), *p* = 0.93]. On an intention to treat analysis, across all 59 participants average CPAP uses was 185 ± 173 min. Of the *n* = 39 who used CPAP during the trial, mean CPAP adherence was 291 ± 126 min, with 27 of these 39 (69%) participants using the treatment on average >4 h per night. Average residual AHI was 2.2 ± 1.9 events/h. Participants were asked at the end of the study period (12 months) if they intended to continue to use CPAP; 33 (77%) reported they planned to continue, 9 (21%) were going to discontinue and one (2%) was undecided.

## Discussion

This study is the first to explore whether timing of commencement of a lifestyle intervention impacts upon weight change and its trajectory over a 12-month period in patients with moderate-severe OSA who are recommended CPAP treatment. This trial purposively utilised the step-wedge design so that no individual was precluded from treatment and so the impact of timing of commencement of the weight loss intervention could be investigated. Our results indicated that participants lost on average 3.7 kgs over the 12-month trial period after exposure to the lifestyle intervention and the timing of when the lifestyle intervention was commenced did not affect either the rate of weight loss or the final weight achieved at 12 months. Commencing CPAP alone did not lead to weight change.

The relationship between body weight and CPAP treatment has been explored previously with some evidence that increased adherence with CPAP results in a modest gain in body weight [20, 21]. A recent review concluded that OSA itself may reinforce the obese state via alterations to energy metabolism, appetite and satiety via neural control feedback mechanisms, thereby making it harder for those with OSA to manage their weight once on CPAP [2]. In this study we were able to explore weight trajectory during the time when participants were using CPAP, but not exposed to a weight management program, and we found that CPAP alone did not increase their body weight. This finding is important as it provides confidence that upon commencing CPAP treatment weight gain is not an inevitable consequence.

This trial was purposively designed to elucidate if there is an optimal window of opportunity to introduce additional new weight management behaviours that are synchronous with the behaviours necessary to undertake CPAP treatment. We found that timing of the commencement of the lifestyle intervention had no impact on weight after 12 months, following exposure to the CPAP and the weight management program. This is important clinical information to guide practitioners in counselling patients who are newly diagnosed with OSA who are also above a healthy weight. The weight loss patterns observed in this trial also supports that the first three months of attempting weight loss is the most impactful period, after which weight loss trajectory slows, and maintenance of lost weight subsequently becomes challenging.

Theoretically, individuals who are successful in changing their behaviour do so by making small incremental steps towards a long-term goal and there are three main factors operating that influence behaviour change; capability, opportunity and motivation [18]. We hypothesised that participants who waited a few months after starting CPAP treatment were more likely to be successful in managing their weight in the longer term as they had time to develop and adopt the behaviour change of CPAP implementation, prior to that of a weight loss intervention. However, our results demonstrate that a waiting period before trying to lose weight does not confer any benefit nor disadvantage. This suggests that if weight loss is clinically indicated, patients newly diagnosed with OSA can be advised to commence a lifestyle-based weight loss program at the same time as starting CPAP or, if they choose to delay weight loss treatment, it is likely they will still confer some benefits.

With healthcare systems actively encouraging and investing in technological innovations to support available routine care [22], this study sought to explore whether providing participants with an App designed to motivate and support achievement of behavioural goals resulted in enhanced engagement with their dietician during a weight loss attempt. Engagement with the App was low with 49% of participants not engaging with the App at all, despite 87% having access to a Smartphone. This may be in part due to the monthly contact and engagement with the study team, and this level of face-to-face support was valued highly by participants in their feedback (see supplementary file). This may reflect the traditional expectations of the participants (mainly mid-aged males) that allied health interaction is delivered face to face.

Overall uptake of CPAP during the study was low, with average daily use across all trial participants of 3 h and 5 min. However, this was a pragmatic trial, likely representing real world CPAP use and as such this uptake is similar to that seen in another recent intervention trial [23]. Of the 39 subjects who used CPAP, average use was 4 h and 51 min and intention to continue with CPAP was similar or higher than other studies, with 80% of completers reporting that they intended to continue [24]. Importantly, there was no difference in weight loss in those who used versus didn’t use CPAP. Issues of adherence with CPAP have been consistently described in a systematic review [4], and highlighted in the 2020 Cochrane review [25]. Although behavioural interventions have been shown to increase CPAP usage, this was not the focus of our study, where the intervention being tested was a lifestyle weight loss intervention.

The style of dietary pattern used in this study, which adopted an intermittent energy restriction approach, was well received, with completers reporting a high intention to continue post-trial. Synthesis of evidence from pre-clinical and clinical studies employing intermittent energy restriction have demonstrated this approach to have broad spectrum benefits [26] and are just as effective in terms of weight loss and reduction in cardiovascular risk than continuous energy restriction [27]. There is no doubt that mean weight loss was modest and potentially withdrawing dietitian support after 6 months in those with significant obesity is premature, but this would require further long term studies.

Strengths of this study include the high level of intervention fidelity achieved and the overall 78% completion rate. Whilst still maintaining statistical power because of the stepped-wedge design, this is lower than trials in OSA population of similar duration [28, 29]. A limitation is the relatively low CPAP adherence at 12 months. However, given the intention to treat approach when data has been analysed from the stepped wedge element, it is likely that the effect sizes reported here are not over-estimates of what could be achieved in real life.

In conclusion, this study demonstrates the benefits of a structured weight management program both in terms of reduction of severity of OSA and a modest reduction in body weight. The timing of starting a weight loss attempt does not impact outcomes after 12 months.

## Supplementary information

Supplementary Information

## Data Availability

The datasets generated and analysed during the current study are available from the corresponding author on reasonable request.
