# Supplementary material for: A 12-month weight loss intervention in adults with obstructive sleep apnoea: is timing important? A step wedge randomised trial
Source: Eur J Clin Nutr. 2022 Aug 4;76(12):1762–9. doi: 10.1038/s41430-022-01184-5 (PMC9708544; doi:10.1038/s41430-022-01184-5)
Supplement: Supplementary file 1 — Supplementary Information [file 41430_2022_1184_MOESM1_ESM.pdf]

Supplementary Information

A 12-month weight loss intervention in adults with obstructive sleep apnoea: is timing important? A randomised trial.

Helen Truby, Bradley A Edwards, Denise M O'Driscoll, Alan Young, Ladan Ghazi, Claire Bristow, Kerryn Roem, Maxine P Bonham, Chiara Murgia, Kaitlin Day, Terry P Haines, Garun S Hamilton

Methods

Polysomnography

Full Polysomnography was performed in an in-patient setting (level 1) or in the patient's home (level 2). The recording included electroencephalogram (EEG), bilateral electrooculogram (EOG), mentalis/submentalis electromyogram (EMG), anterior tibialis (left and right) EMG and electrocardiogram (ECG). Respiration was assessed via nasal pressure cannula +/- oronasal thermistor, thoracic and abdominal respiratory inductance plethysmography (RIP) bands, and fingertip pulse oximetry.

Sleep stages, arousal, and respiratory events were scored according to American Academy of Sleep Medicine (AASM) 2012 recommended criteria using Profusion PSG3 software (Compumedics, Abbotsford, Victoria, Australia) (1). Specifically, AHI was defined as the total number of apnoea's and hypopneas per hour of sleep. Apnoea was defined as a ≥90% decrease in oronasal airflow for at least 10 seconds. Hypopneas were scored when nasal pressure signal dropped by ≥30% from baseline for ≥10 seconds and the event was associated with either a 3% or greater fall in oxygen and/or an arousal from sleep.

Intervention

One-to-one appointments with the study dietician (KR) were arranged monthly for the duration of the 6-month intervention which Months 1-3 focused on active weight loss, whilst months 4-6 focused on strategies to maintain lost weight. Participants were offered the use of a smartphone app (MyPace™) for communication with the dietician in between their monthly face-to-face appointments. On this app, participants could set and monitor their progress towards their own goals and track their weight loss. Motivational texts were sent to the participant at times that were agreed between the dietician and the participant and two-way communication with the dietitian was possible for additional support in between consultations. Participants were advised to increase their physical activity levels (i.e. 3 x 30 minutes/week moderate activity such as walking or swimming) by the dietician at their first appointment and were provided with a Fitbit© to help participants' monitor their own physical activity levels. Participants provided separate consent for the research team to access their individual web-based FitBit© account to monitor usage and activity.

Supplementary Tables

Further analysis and results of primary aim 2: investigation of the effect of timing of commencement of the lifestyle intervention of weight outcomes.

Effect of timing of the intervention was investigated using multilevel, mixed effects, generalised linear models using combined data from the stepped-wedge portion of the study design and follow-up data using intervention-by-month of commencement, as a categorical variable, as an interaction effect. In addition, this aim will be investigated using ANCOVA-style linear regression analysis. This analysis utilises weight as a raw score as the dependent variable and month of commencement of the intervention is treated as a categorical variable. The first weight measurement taken (baseline weight) as a raw score is used as a covariate.

As the initial analysis (supplementary table 1) revealed that commencement of the intervention at month 4 trended towards significance ($p = 0.05$) further analysis was undertaken to investigate this. This included changing the reference month to month 4 rather than month 1 and a quadratic transformation with month 4 as the peak (supplementary table 2). Neither further analysis revealed any significant result. Finally, it was further investigated if timing of commencement of the lifestyle intervention has an effect on weight outcomes at 12-months, again, this revealed no significant interactions (supplementary table 3).

Table E1. Effect of timing of commencement on weight change (note: Month 1 is referent month).

	Coefficient	95% CI	P value
Month 2	-0.47	-2.00 to 1.91	0.96
Month 3	0.79	-0.71 to 2.29	0.30
Month 4	1.60	-0.00 to 3.19	0.05
Month 5	0.53	-0.98 to 2.04	0.49
Month 6	0.59	-0.89 to 2.07	0.44

Table E2. Data transformations to explore effect of timing of commencement on weight change (note: Month 4 is reference month).

	Coefficient	95% CI	P value
Month 1	-0.80	-2.14 to 0.53	0.24
Month 2	-1.64	-3.48 to 0.19	0.08
Month 3	-1.60	-3.19 to 0.00	0.05
Month 5	-1.07	-2.42 to 0.29	0.12
Month 6	-1.01	-2.33 to 0.31	0.13
Quadratic transformation of linear variable month of commencement with month 4 as peak			
Month	-0.11	-0.26 to 0.04	0.14

Table E3. Effect of timing of commencement on 12-month weight outcomes (note: Month 1 is referent).

	Coefficient	95% CI	P value
Month 2	2.76	-4.73 to 10.25	0.46
Month 3	3.35	-3.52 to 10.21	0.33
Month 4	-3.42	-11.43 to 4.59	0.39
Month 5	-0.86	-8.09 to 6.36	0.81
Month 6	-1.32	-8.32 to 5.69	0.71

Process Evaluation

A mixed-methods process evaluation was implemented. Michie's Behaviour Change Taxonomy was applied throughout in order to provide structured support and monitoring methods. Participants' were provided with an end-of-trial feedback form in order to gain insights into their experiences and perspectives of the study, completed on an iPad. This procedure enabled both qualitative and quantitative analysis of the different aspects of the trial to be analysed.

Intervention

Participants were required to attend an appointment every month for the duration of the trial. Some months required more than one appointment, depending on which phase of the trial the participant was in (for example month six where participants visited the dietitian and Air Liquide™ in the same month). In total, there were 16 compulsory scheduled visits from enrolment to conclusion of the trial (excluding initial pick-up and final drop-off of the CPAP machine which were organised in the participant's own time) (research centre, 7 visits, Air Liquide™ centre 3 visits and dietitian consultations 6 visits). Throughout the trial,

participants were made aware that staff members from all centres were available to provide support/troubleshooting when needed, and additional appointments could be arranged at the research and Air Liquide™ centres if necessary. Trial staff were also available via telephone during business hours.

Intervention dose overall was high, with the median number of sessions attended 14 out of 16 (87.5%) (dietitian 6 out of 6 (100%), Air Liquide™ 1 out of 3 (33%) and research centre 7 out of 7 (100%)). Figure E1 presents an overview of participant monthly attendance over the trial period. Intervention fidelity, the extent to which the intervention was delivered as planned was moderate-to- high, with the intervention protocol being delivered successfully in accordance with the protocol developed pre-trial. Exceptions existed when participants' developed conditions in which variances in procedures needed to occur, i.e. developing type 2 diabetes, irritable bowel syndrome (IBS) or sustaining an injury. In such instances, other arrangements were made (e.g. follow a low FODMAP diet whilst maintaining two fasting days, practice rehabilitation exercises) whilst trying to adhere to the original protocol as closely as possible.

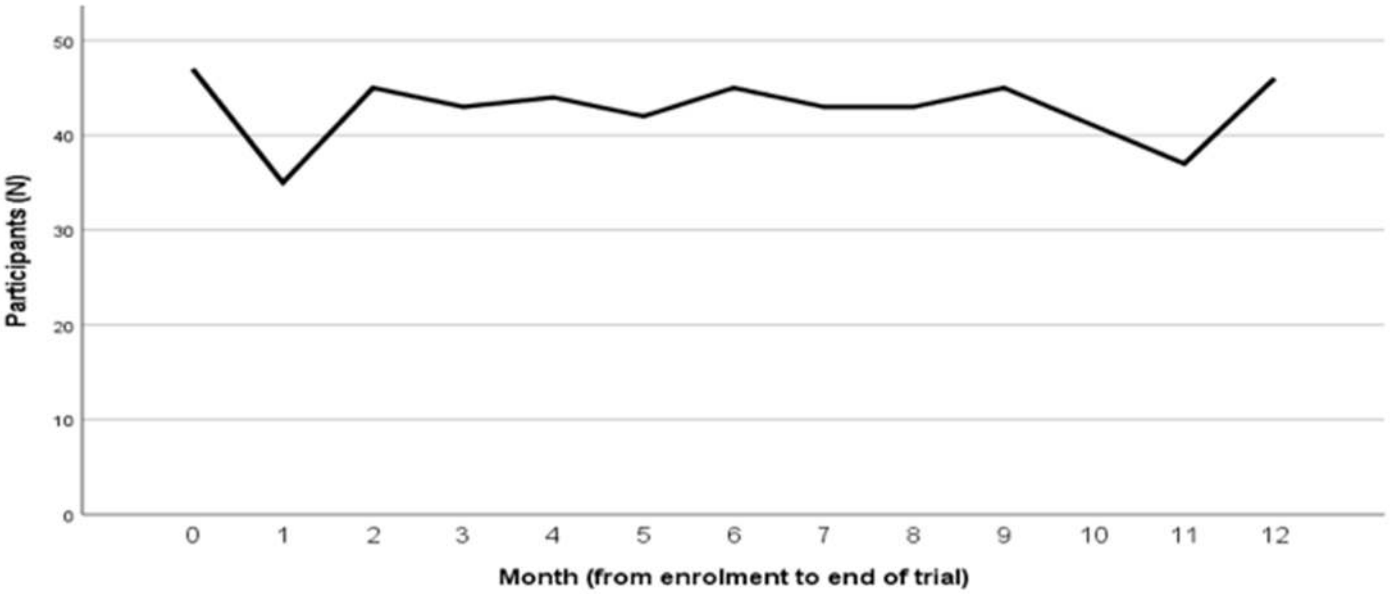

Figure E1. Participant monthly attendance (n 47) Figure 1 demonstrates the number of participants (Y-axis) attending their designated session for the corresponding month (x-axis).

The perspectives and experiences of completers with specific aspects of the trial procedures.

App usage

Forty-one out of 47 completers had the MyPace™ app installed on their smartphone devices. The remaining participants did not have a smart device on which the app could be installed. Usage could be classified into two categories; one-way (single message from one party) or two-way interaction (engagement between both parties). Twenty-three (49%) of participants did not interact at all with the dietician at all using myPace™, hence dose for this particular aspect of the trial was low. Women were more likely to engage with the app than men ($p= 0.006$). User ratings are presented in table E4.

Physical activity and Fitbit©

The median number of months in which participants used the Fitbit© device (defined as at least one recording present on a participant's online accounts) was 10 months (IQR 5, 12). Months four, nine and ten were the most frequent months in which usage of the device ceased. Participants over 50 years of age engaged with the device more than those under 50 years ($p<0.001$). User ratings are presented in table E4.

Table E4. Competing participant (n=46) ratings of individual components of lifestyle intervention on a 5-point Likert scale from 1 (not very useful) to 5 (extremely useful) and stated intentions to continue post-trial

**Usefulness rating**	**Median**	**IQR**
myPace™	2.0	(1.0, 3.0)
Fitbit©	4.0	(3.0, 5.0)
Dietician sessions	5.0	(4.0, 5.0)
	**Yes**	**No**
Intention to continue lifestyle intervention after end of dietitian sessions (n, %)	43 (91.5)	3 (6.4)

Participants' opinions about the intermittent energy restriction were overwhelmingly positive, with particular emphasis on the role of the dietitian throughout.

P08- "Highly recommend. [Dietician's name] is just great"

P59- "Very easy to stick to. Dietician very helpful in explaining what works/how"

The few people who struggled with the intermittent fasting days indicated it was mainly timing that hindered their success and not the program itself.

P25- "It was good, but unfortunately life got in the way"

P45- "A good idea, good plan, but being a rostered shift worker…very hard to follow"

Only one participant did not favour the intermittent energy restriction as they believed it made them focus on food too much, which had never previously been an issue for them.

P55- "Made me think more about food/eating than before- previously food was not on my mind"

Participants' ratings of the dietician sessions are presented in table E4, as well as whether or not they intend to continue the lifestyle intervention post-trial. For those who answered yes, diet changes alone or in combination with exercise, opposed to solely exercise, were the most commonly discussed strategies. Intended dietary changes generally included an increase in fruit and/or vegetables, decreased portion sizes or specifically continuing with the intermittent energy restriction dietary pattern.

P01- "Restrict the amount of fat intake and increase vegetables and fruit"

P07- "Less carbs and more [veggies]; learn to say "no more often to extra food"

P15- "Still train and do the 5:2 diet [until] I hit my goals"

P26- "Control meal size/snack type and size and keep walking

CPAP

Two main themes emerged when participants were asked to explain why they intended to continue/discontinue CPAP post-trial. The first, and most commonly discussed, was that the

machine provided them with some sort of relief and/or benefit (i.e. better sleep, more energy) thus leading to an overall improvement in quality of life. The second was that there was a belief they needed it for their health and to reduce risk of future illness.

P32- "Makes a [huge difference] in the quality of sleep. Without it I wake up with a headache and not as well rested"

P06- "I need to, to stop snoring"

P07- "Dr has said it will lessen my risk of heart attack and stroke"

Similarly, in those who opted not to continue, two predominant reasons were present; participants disliked the machine (inconvenient/uncomfortable) or they believed they no longer needed to use it.

P37- "It was too noisy for my kids and there are blisters formed on my face as a result of using it. It was painful"

P26- "My condition was improved enormously- however if I do [require] to use again I will"

Participants were asked to then give further detail as to the main barriers of CPAP usage, as well as any recommendations for implementing CPAP in future studies. Issues with the mask (an attachment on the machine) were very clearly emphasised as the strongest barrier.

P01- "Getting the right mask at the start and working out how to set up the mask properly was a bit problematic"

P57- "Finding the right mask was the biggest issue"

Inconvenience of the machine either when sick or travelling also emerged.

P06- "Travel- bulky and heavy"

P44- "1. When I was travelling. 2. If I have [a] sore throat…"

P58- "When I have a cold…"

That participants' were considering the impact of CPAP not only on themselves but also their families was evident.

P37- "It was too noisy for my kids" "1. Kids complain about noise"

P09- "Have a young baby to take care [of] and have to wake up several times during [the] night"

P18- "Impact on my wife due to noise"

Few recommendations for future studies were provided, with many participants' indicating they were satisfied with CPAP overall. Suggestions that did arise centred on allowing participants' adequate time to adjust to the machine as well as providing them with more information.

P01- "Make sure the participant takes some time to get the set-up right"

P02- "Need to get the mask working well before starting…"

P22- "Participant needs to understand more why using CPAP machine, more explanations?"

Reducing participant burden was also suggested.

P52- "It would be good if adjustments could be made during visits to BASE"

P58- "It would be good if there was not much that you have to organise"

Further, we asked participants to share particular aspects of the trial they found helpful/unhelpful. Support and commitment came across as an important aspect for many people.

P02- "Commitment. As it was a study I [persevered]"

P23- "All the staff made me feel very worthy and they cared about my health. They [were] very very helpful and polite"

P48- "Very well supported the whole way through"

The dietician sessions and lifestyle intervention were also frequently described as helpful, with only one participant feeling there were too many sessions.

P01- "…diet intervention very helpful"

P07- "Helpful to have input from the dietitian and the fasting technique"

P52- "Too many dietician visits"

Finally, participants were asked about data collection methods throughout the trial and whether they had any suggestions on how they can be improved. This was the least answered question, however of those that did respond, many complimented the way the trial was run and praised the study overall.

P32- "Don't change anything"

P51- "All good"

The few suggestions given to improve data collection were focused around participants' being provided with more feedback.

P02- "…would like more feedback on results"

P38- "All good. Feedback on the [fitness] results"

References:

1. Berry RB BR, Gamaldo CE, Harding SM, Marcus C,, B V. *The AASM Manual for the Scoring of Sleep and Associated Events. Rules, Terminology and Technical Specifications. The AASM Manual for the Scoring of Sleep and Associated Events. Rules, Terminology and Technical Specifications.* American Academy of Sleep Medicine: Darien; 2012.
